# Correlation Characterization of Particles in Volume Based on Peak-to-Basement Ratio

**DOI:** 10.1038/srep43840

**Published:** 2017-03-02

**Authors:** Tatiana A. Vovk, Nikolay V. Petrov

**Affiliations:** 1 ITMO University, Department of Photonics and Optical Information Technologies, Saint-Petersburg, 199034, Russia

## Abstract

We propose a new express method of the correlation characterization of the particles suspended in the volume of optically transparent medium. It utilizes inline digital holography technique for obtaining two images of the adjacent layers from the investigated volume with subsequent matching of the cross-correlation function peak-to-basement ratio calculated for these images. After preliminary calibration via numerical simulation, the proposed method allows one to quickly distinguish parameters of the particle distribution and evaluate their concentration. The experimental verification was carried out for the two types of physical suspensions. Our method can be applied in environmental and biological research, which includes analyzing tools in flow cytometry devices, express characterization of particles and biological cells in air and water media, and various technical tasks, e.g. the study of scattering objects or rapid determination of cutting tool conditions in mechanisms.

The studies of parameters of particles in suspensions and flows are necessary in various branches of science. Phytoplankton analysis^1^, aerosol distribution in the atmosphere^2^,^3^, airflow measurements^4^ microfluidics^5^, two and combined phase flows^6^, spray and combustion characterization^7^, monitoring of cavitation appearance^8^, and virus tracking in live cells^9^ are only few on hearing examples.

There are many imaging techniques for investigation of various particles properties, as well as methods for processing of their images. For instance, to measure the particle velocities in flows Particle Image Velocimetry (PIV) used as a rule^10^. Depending on the task conditions, various modifications of the PIV technique can be implemented, as micro-scale^11^,^12^ or micron-resolution^13^, holographic^14^ and tomographic^15^ extensions. Various automated microscopic image analysis techniques are used for particle size and shape characterization^16^. But the problem of limited imaging depth of field restricts their capabilities. One of the possible solutions are the Digital Holography (DH) methods, which are widely used^14^,^17^, since they provide the possibility of numerical post-process image refocusing, so three-dimensional profiling and particle tracking become possible. In the case of coherent illumination the procedure of focusing in particular plane can be carried out by means of numerical calculation of the diffraction integral, which describes the wavefront propagation. Electromagnetic energy is distributed non-uniformly as diffraction spots and artifacts. It forms an image of particle by focusing in a predetermined plane. The particles in the image plane that was focused initially are converted into an inhomogeneous pattern again. In this context, the problem of particle profiling occurs^18^,^19^. In the case of frame series that describe particle dynamics, there is an appearance of the 3D tracking task^9^,^20^,^21^. Mostly the concentration of investigated particles is small since the inhomogeneous illumination for high particle concentrations forms a coherent speckle field, greatly limiting the possibility of particle recognition. Despite the presence of incoherent illumination formed by the flow of high-temperature supersonic particles, detection of objects is only possible with high-speed electro-optical shutter cameras^22^.

Our previous works^23^,^24^ describe a special method for determination of particle concentrations based on edge-point linking and threshold processing. The method had shown a good performance at high concentrations of particles, however it had quite large computational complexity.

Here we consider the problem of fast estimation of the particles’ concentration in the volume. In this paper we introduce a new method of correlation analysis in order to accelerate the image processing. In digital processing the calculation of the cross-correlation (CC) function has undeniably high speed. This advantage is used in many studies that require obtaining the instantaneous information related to rapidly changing environmental conditions, for example when measuring shifts^25^,^26^, deformations^27^, the surface roughness^28^, and for plotting of the vector field of objects velocities^29^,^30^. Given research involves the calculation of the peak-to-basement ratio (*p*/*b*) of the CC function. After preliminary calibration by numerical simulation, this ratio allows fast and reliable evaluation of the particle suspension parameters. We consider scattering particles of a fixed size and spherical shape which are distributed in the volume of the optically transparent medium. For given conditions we propose express method for particle concentration study via CC function calculation. We describe the sequence of method application, investigate the dependency of the CC function on particle parameters, and evaluate the effectiveness of proposed approach in experimental model conditions. Further we discuss the applications of the proposed method in biological and technological research.

The paper is organized as follows. In Results and Discussion section investigations of the method and application of the algorithm are shown. The dependency of CC function on variation of single parameter is shown in General consideration of CC function dependencies through the numerical simulation subsection. A detailed consideration of mutual contribution of several parameters is presented in CC function dependency on several parameters subsection. In Experimental validation subsection the results of experimental verification are shown. In Discussion subsection the interrelation of conducted experiments and practical problems are discussed focusing on the specific areas where the method can be demanded. In Methods section we describe all the theoretical aspects related to the proposed method: General description of technique subsection defines the task and the ‘geometry’ of the numerical simulation; Single particle characterization, Distribution of particles and Wavefront propagation model subsections provide description of particle appearance, the concepts of particles allocation in the volume, and numerical method for wavefront propagation, respectively; Image processing subsection gives the theoretical aspects of CC function calculation that was used for evaluation of the particle concentration.

## Results and Discussion

### General consideration of CC function dependencies through the numerical simulation

**Figure 1:**
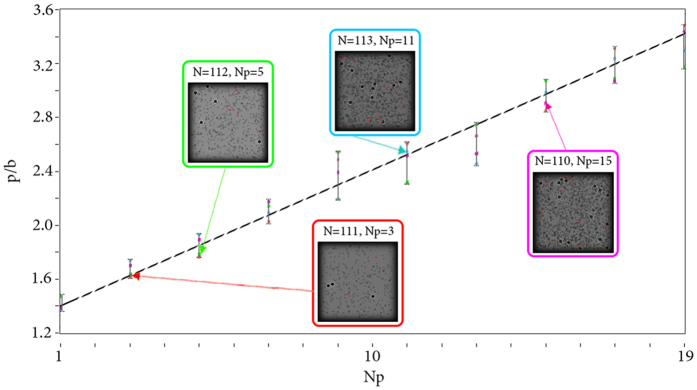
Dependence of *p*/*b* on the normalized concentration *C*_*n*_ of the particles in the volume. Footnotes indicate the particle distributions, for which the CC function is calculated. *D* = 0.054 mm, Δ*l* = 0.054 mm, *T* = 0%. Correlations are calculated for the first and second layers. Screen sizes 3.072 × 3.072 *mm*^2^, *λ* = 632.8 nm.

**Figure 2:**
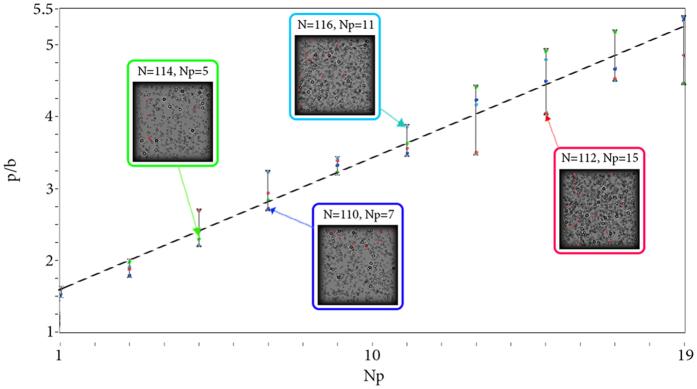
Dependence of *p*/*b* on the normalized concentration *C*_*n*_ of the particles in the volume. Footnotes indicate the particle distributions, for which the CC function is calculated. *D* = 0.072 mm, Δ*l* = 0.072 mm, *T* = 0%. Correlations are calculated for the first and second layers. Screen sizes 3.072 × 3.072 *mm*^2^, *λ* = 632.8 nm.

We have considered different cases of particle distributions and observed the dependency of CC function on such parameters as transparency, sizes, etc., according to our model. For the proposed model all of the dependencies will be approximately linear. Consideration of various cases of random particle distribution in the volume is shown in Figs 1 and 2.

The dashed line in each of Figs 1 and 2 represents an approximation of the dependencies. In these figures, we varied the size of the particles leaving other parameters unchanged. But the whole system of such dependencies is flexible, and other parameters of particles can be changed forming other relation databases. With the diameter increase the Arago spot appears (Fig. 2). However, the general dependency is still linear.

**Figure 3:**
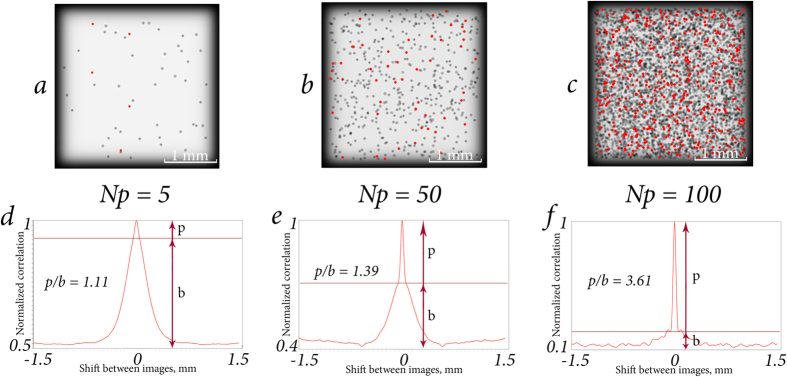
Particle suspensions (**a**–**c**) with Δ*l* = 0.15 mm, *D* = 0.054 mm, *N* = 10, *T* = 0% and CC functions (**d**–**f**), calculated for the first and second layers. Other parameters: as in previous figures. The focused particles are marked with red rounds. *p*/*b* ratio is growing with the increase of normalized concentration.

**Figure 4:**
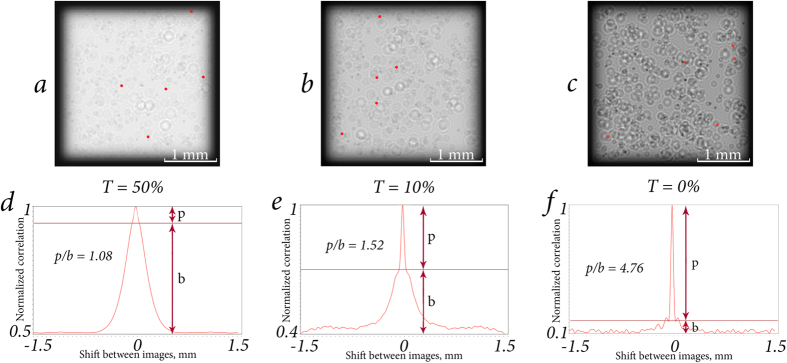
Particle suspensions (**a**–**c**) with Δ*l* = 0.15 mm, *N* = 100, *N*_*p*_ = 5 and their CC functions (**d**–**f**), calculated for the first and second layers. Other parameters: as in previous figures. The focused particles are marked with red rounds. With the decrease of the particles transparency, *p*/*b* ratio also grows and the peak stands out from the basement.

**Figure 5:**
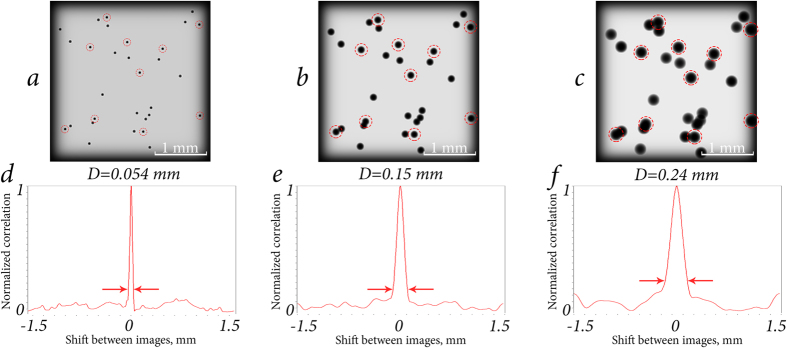
Particle suspensions (**a**–**c**) with Δ*l* = 0.15 mm, *N* = 3, *N*_*p*_ = 10, T = 0% and their CC functions (**d**–**f**), calculated for the first and second layers. Other parameters: as in previous figures. The focused particles are marked with red circles. The peak is broadening due to an increase of the particle size.

An elementary consideration of other dependencies is presented in Figs 3, 4 and 5. Recording such dependencies in the general database, it is possible to create a hardware-software system that will count particle concentrations in the optically transparent medium. In addition, formation and maintaining of a large database of speckle patterns is successfully used in similar methods^31^.

CC function of two focused layers of particle distribution depends on many parameters. Among them are the particles’ diameter *D*, number of particles per layer of volume (or normalized concentration, *C*_*n*_ = *N*_*p*_), wavelength of the laser source *λ*, transparency of particles *T* (see definition in Single particle parametrization subsection, Methods section), etc. Examples of the impact of some of these parameters are shown in Figs 3, 4 and 5. Dependencies shown in Figs 3 and 4 are quite similar due to the fact that the CC function is very sensitive to repetitive patterns of the images. The CC function allows to extract information about the differences between the focused and defocused particles. Thus, defocused particles contribute to the basement of the CC function. With the concentration increase (Fig. 3) or transparency decrease (Fig. 4), the number of unique elements of the overall image grows. Consequently, the basement of CC function droops compared with the peak. Dependence in Fig. 5 shows how CC function responds to the increase of the size of identical elements, namely the spherical particles. It is known that when CC function calculation is performed, the correspondences between two functions in one shift step relative to each other are considered. The larger is the particle, the greater is the shift step that will cease the correspondence. That is the reason why the peak is broadening while the size of the particles is increasing.

### CC function dependency on several parameters

In addition to the above let us consider the case when the effects of several characteristics are mixed. In such configuration the CC function shape dependencies are no longer as simple as in previous subsection. However, among this great set of effects it is possible to identify the main subset in which the monotonic dependencies are observed. In this subset CC function behaviour can be extrapolated.

Consider the trivial case when the diameter of particle is growing with the increase of concentration. This type of problem is similar to more rapid concentration increase with constant diameter. That is, with the growth of these two parameters particle diameter rise will contribute to the concentration growth, because diameter rise can act as formation of agglomerated particles.

The diameter decrease with the rise of concentration is more interesting task. In such case parameter variation does not conduct to the mutual enhancement, and these parameters are presented as independent from one another. Growing diameter of particle does not amplify the rise of concentration. Increasing concentration continues to amplify the peak-to-basement ratio, and decreasing diameter narrows the CC function peak while *p*/*b* ratio remains the same. Table 1 shows the average *p*/*b* ratio values for various diameters and concentration of particles.

The mutual increase of the particle opacity and concentration also results in a simple dependency. This task is close to the one shown in Table 1, but with the transparency rate being the source of the concentration growth amplification. At that, the increase of opacity should be interpreted as the overlapping of translucent particles, which means the additional growth of the concentration.

What is more interesting is the case of simultaneous concentration and transparency growth. Mutual enhancement is not observed here as well. The transparency growth contributes to the pedestal of CC function since the particles are harder to distinguish from the background illumination. Hence, even with the increase of concentration, *p*/*b* ratio grows down. Table 2 shows the average *p*/*b* ratio values for various concentration and transparency rate of particles.

It is also necessary to consider the case of the simultaneous variation of the particle diameter and transparency. With the increase of diameter and opacity of the particles, the growth of *p*/*b* ratio takes place since this mutual effect can be interpreted as the increase of the concentration of translucent particles. In the opposite situation the transparency growth causes the drop of this ratio since transparent particles are harder to distinguish from the background. Table 3 shows the average *p*/*b* ratio values for different diameter and transparency of particles.

**Figure 6:**
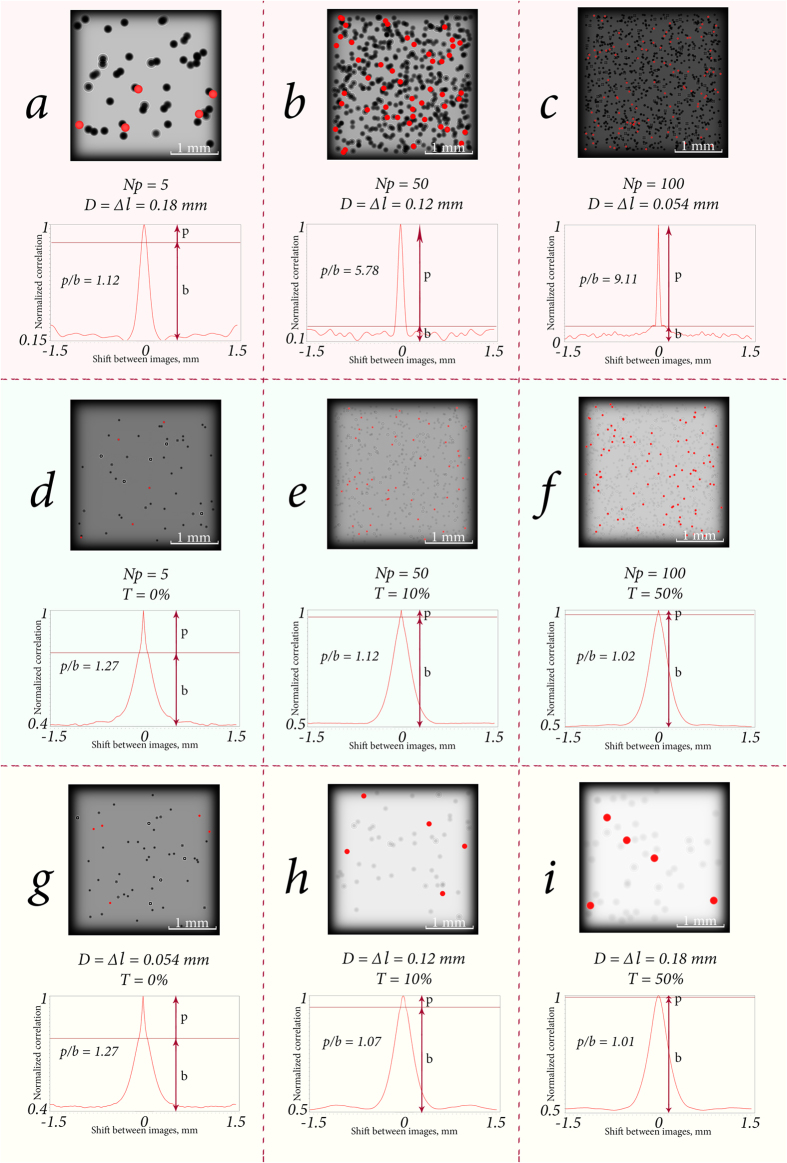
Particle suspensions and their CC functions with mutual variation of diameter and concentration (**a**–**c**), concentration and transparency (**d**–**f**), and transparency and diameter (**g**–**i**). The focused particles are marked with red rounds.

The most interesting dependencies of considered cases are shown in Fig. 6. On the basis of *p*/*b* ratio values in the Tables and on Fig. 6, several conclusions can be drawn. With increase of particle transparency, diffraction patterns become less distinguishable from the background illumination, which leads eventually to a quasi constant parameter value that is close to 1 (see Fig. 4, Tables 2 and 3). It means that, with increase of opaqueness, the concentration and the diameter of the particles are better discernible. From Table 1 one can see that with concentration and diameter variation, the concentration has the biggest influence on *p*/*b* ratio. In the case of various concentration and transparency, influence of concentration on *p*/*b* ratio decreases with increasing transparency (Table 2). Likewise, the diameter impact is reduced with the decrease of opaqueness (Table 3).

In summary for given subsection, we have performed analysis on the effect of the following mixed parameters on the CC function peak-to-basement ratio: particle concentration represented via number of particles in one layer *N*_*p*_, particle diameter *D* and transparency *T*. It was found that the concentration has the significant influence on *p*/*b* ratio while the other parameters make not so strong difference in CC function when the parameters are mixed. The increase of the particle diameter provides the similar effect on the CC function peak-to-basement ratio as the augment of *N*_*p*_. It can be interpreted as the raise of concentration in terms of overall particle density. The variation of the transparency makes less tangible effect when the parameters are mixed, and CC function is not able to reliably distinguish this variation. That is why in given paper we primarily focus on the construction of the method for particle concentration characterization. But subsequently it can be configured for different parameter variations, if necessary.

### Experimental validation

**Figure 7:**
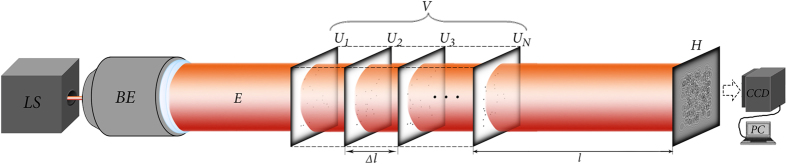
Scheme of the physical validation. *LS* – laser source, *BE* - beam expander, *E* - plane field from the source, *V* – test object, consists of *U*_*i*_ - layers, *H* - received hologram, *CCD* – photodetector, *PC* - computer.

We have also conducted several experiments in order to test our assumptions in real conditions. First of these was a static experiment, i.e. there was a completely stationary object under investigation. Second experiment showed the dynamic type of the possible application, that is, there was a non-stationary object. Both of the experiments were performed according to the same general scheme, shown in Fig. 7.

**Figure 8:**
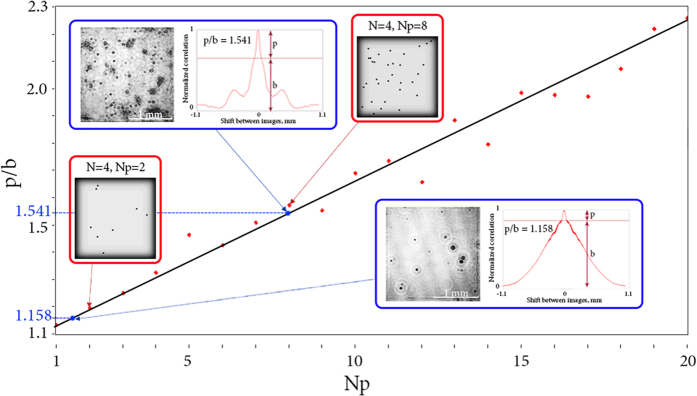
Simulation of real conditions (red dots and approximation black line) with the experimental results (blue dots) marked on it. Simulated objects are shown in red footnotes. Objects and cross-section of CC functions are shown in blue footnotes. Typical particle size 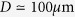; Δ*l* = 1.5 mm; *T* = 0%; *N* = 4; *D*_*x*_ × *D*_*y*_ = 2.151 × 2.151 *mm*^2^; *λ* = 532 nm.

In the first experiment the target region consisted of a set of glass slides spaced apart at equal distances. Each slide was randomly coated with an opaque paint. Parameters of the experiment: laser source Lasos GLK-532-20-0,7-2 with *λ* = 532 nm; CMOS matrix VEI - 830, 800 × 600 pixels, Δ*x* = 2.8 *μ*m (pixel size). Experimental results are shown in Fig. 8.

While conducting this validation, it was difficult to consider all features of the proposed model which affected the results in a particular way. Firstly, there was a small number of layers with particles and a great distance between them compared to the average particle size. Secondly, due to the randomness of paint distribution on glass slides, sizes of each ‘particle’ were slightly different. That is why there is an inaccuracy for number of particles in each layer. Despite that, this experiment is a real embodiment of the considered model, even if it does not consider some of its features. And through this test it was found that the proposed method is able to distinguish different concentrations of particle samples.

In the second validation the object was a suspension of small iron filings in viscous liquid in a cuvette. In this experiment, the layers of the volume approached each other at the minimal distance, and two-phase flows of different types of particles contributed additional errors. Parameters of the experiment: laser with output power 5 mW and *λ* = 650 nm; for inline holograms registration CMOS Nikon D3100 with 4608 × 3072 pixels and pixel size of Δ*x* = 5 *μ*m was used; longitudinal size of the cuvette was 10 mm.

**Figure 9:**
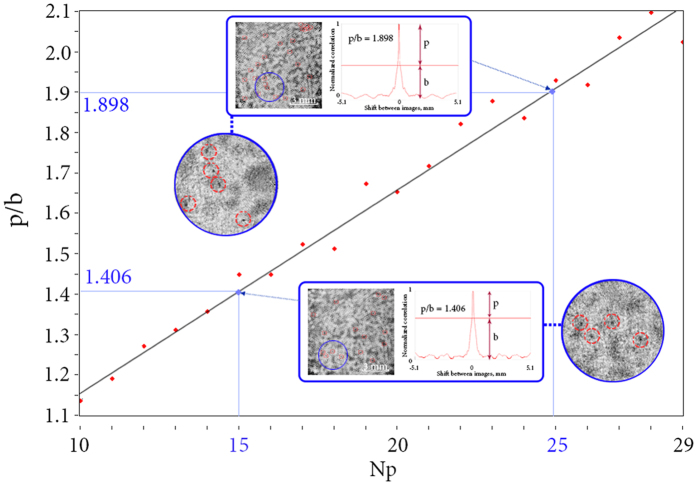
Simulation of real conditions (red dots and approximation black line) with the experimental results (blue dots) marked on it. Simulated objects are shown in red footnotes. Objects and cross-section of CC functions are shown in blue footnotes. Enlarged images of the objects are shown in blue circles. The focused particles are marked with red circles. Particles are considered to be completely opaque, typical particle size 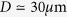; Δ*l* = 30 *μ*m; *T* = 5%; 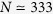; *D*_*x*_ × *D*_*y*_ = 10.12 × 10.12 *mm*^2^; *λ* = 650 nm. *N* has been chosen based on the cuvette size and the volume partition rank.

In this experiment, the particles could be divided into two types, namely, small light fraction of particles and massive particles of larger diameter. Light particles remained floating in the liquid, and were closer to the camera, while the particles of the second type have sunk mostly remaining at the background. This effect was caused by the peculiarities of the procedure of the particles’ supplementation into the viscous fluid during the experiment. Results are shown in Fig. 9.

Unlike the first experiment, in this case the whole volume was occupied by the particles without voids along the longitudinal coordinate. It means that the model parameter Δ*l* is equal to the average diameter *D* of the particles. As mentioned in the Distribution of particles subsection of the Methods section, errors may occur in the case of smaller Δ*l*. Despite the large amount of noise, as well as the small particle size, the proposed method had distinguished various concentrations of randomly distributed particles.

Both physical experiments had demonstrated compatibility with the numerical dependencies built for similar conditions. Therefore, they confirmed our assumption of linear dependence of the *p*/*b* parameter on the normalized concentration of particles.

## Discussion

The proposed method is targeted at fast measurement and express evaluation of the concentration of particles suspended in the volume. Let us consider the connection of our experiments with the actual tasks and outline several possible areas where the method could be in demand.

The stack of glasses with randomly arranged paint particles inside as in the first experiment is an example of multilayered transparent object. The dynamic monitoring of the simple layered structure can form the basis for variety of technical applications of the proposed method, especially when the rapid evaluation is required. The general concept of such applications can be divided into three steps. Firstly, one performs the characterization of multilayered object. Secondly, one compares it with the valid value. Finally, one makes a decision on what type of concentration is considered, e.g. degree of impurity, defects of density or other parameter of materials and products depending on the type of manufacturing appliance. In addition, in the forthcoming studies and development of wavefront shaping techniques targeted on the minimization of the scattering process in disordered media, we intend to apply the developed technique for the characterization of scattering phantoms, constructed from the stack of scattering planes^32^. Conversely, another problem may be of interest, namely the use of this study for the rapid assessment of the quality of three-dimensional image formed by the diffractive optical elements, calculated using the wavefront shaping techniques.

In the case of stirring particles as in the second experiment, the range of applications is quite wide. Suspensions of metal shavings in friction engines and other mechanisms can act as a research object, which appear due to natural causes, such as corrosion, deformation and fibration. This issue is relevant because appeared shavings at sufficiently high concentrations can lead to overheating and equipment damage. In addition, considered case is similar to the task of investigation of colloidal solution of metal chips. Consequently, a problem of rapid determination of the concentration of shavings in the mechanisms occurs. For instance, authors of the paper^33^ state that it would be useful to develop the system for monitoring of cutting tool conditions such as chipping or fracture and thus recognize tool failures by identifying tool states from experience. The proposed method can solve this problem since it is not resource-intensive and therefore may be performed along with other inspection work on the equipment.

Let us also consider several specific applications for the proposed method in the life sciences. Presented developments can be implemented in the environmental studies of the air and water media. The first one is related to the task of express characterization of droplet clusters and ice crystals in the clouds *in situ*^34^. The interest in this problem is driven by the their spatial correlations^35^ in connection with the microphysical parameters of the clouds. The second environmental application in the aqueous media may be associated with the estimation of phyto- and zooplankton density at the oceanic and maritime monitoring^36^. Despite the fact that visual microscopy analysis is an unsurpassed approach for biodiversity studies^37^, it is flawed to provide enough number of data points, which leads to the statistical uncertainty. Contrary, inline holographic observations *in situ*^14^ and *in vitro*^38^ can be considered as more advanced microscopic approach since it allows simultaneous recording of volumetric data and further detailed investigation. In this context, the proposed technique can perform rapid analysis of the real-time captured holograms and capable to replace a tedious, labor intensive and time consuming processing.

In addition, the proposed method can be used in the flow cytometry device as one of the analysis components. This device allows to investigate the suspension of living biological cells^39^,^40^. The principle of flow cytometry method is based on the registration of light scattering from each individual cell in the suspension. The suspension of cells is supplied to the cell flow under pressure. Due to the pressure difference between cell samples and flowing fluid the hydrodynamic focusing occurs: the cells pass through a laser beam one by one. The detectors record the scattering of laser radiation from each cell and transmit this signal to the computer. When using this device, many researchers use sorting of the test cell samples technique. Our express method allows to instantly recognize a cell concentration while using the sorting technique. Thereby it is possible to directly recognize the ratio between different cell types in the sample suspensions.

## Methods

### General description of technique

**Figure 10:**
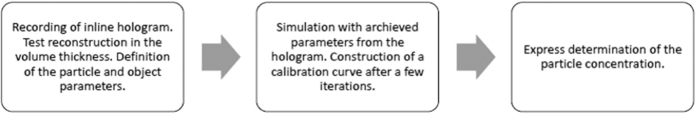
Sequence of processing steps of the proposed method.

**Figure 11:**
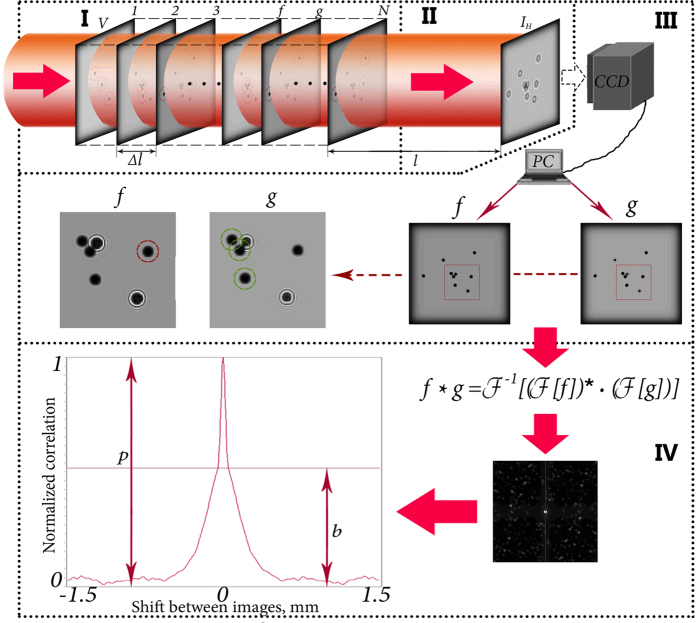
The necessary steps to determine the concentration of particles in the volume using the proposed method. (i) Wavefront propagation through the volume (*V*) with particles uniformly distributed in layers (1, 2, 3, …, *f, g*, …, *N*); (ii) is an inline hologram formation (*H*) in the registration plane; (iii) is a wavefront back propagation to obtain two images of the adjacent layers with particles (*f* and *g*); (iv) is a calculation of the CC function for *f* and *g* and an estimation of the peak (*p*) to basement (*b*) ratio on the cross-section of the CC function.

For successful operation of proposed express method of particle concentration study a preliminary calibration is required. Description of the method application sequence is highlighted in Fig. 10. To perform this we model the volume of the particles in the optically transparent medium. The volume is represented as a set of layers with equal distances maintained between them. Whereby these layers contain particles. The wavefront passing through the predetermined volume is thus scattered by the particles in each layer. Inline hologram is recorded by digital sensor and reconstructed by numerical back propagation of the wavefront to the first layer of volume. Then two adjacent layers with particles are obtained. More accurate focusing can be achieved using any automatic focusing method^41^,^42^. These layers are subsequently compared using the CC function. We analyze the central section of the resulting CC function (explanation of this effect in Image processing subsection). Analyzing the resulting two-dimensional graph, we calculate the ratio of the CC function peak to its basement. On the basis of the ratio value we estimate the concentration of particles with certain parameters, such as the transparency, their size, the distance between the layers with particles, etc. The algorithm is shown in Fig. 11.

However our method is able to operate without prior calibration. In this case it is only possible to determine the relative concentrations of particle distributions. For the case of two distributions it is possible to find the distribution with highest concentration. Several suspensions of particles of the same diameter and different concentration can be sorted in ascending order. In terms of originally proposed ‘calibration’ method it means that instead of simulated base dependencies we can build the real dependency based on experimental data.

### Distribution of particles

The average concentration of common particles in the volume is the ratio of their number to the volume which they occupy. Taking into consideration a predetermined amount of particles in the layer and the number of layers, it is possible to find the value of the concentration. By this definition, the total concentration of particles in the volume is:


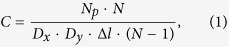


Here Δ*l* is the distance between two adjacent layers, covering only one layer with particles, *N*_*p*_ is the number of particles in one layer of the volume, *D*_*x*_, *D*_*y*_, Δ*l* · (*N* − 1) are linear dimensions of the given volume and *N* is a total number of layers. Thereafter the concentration of particles in the volume is proportional to the expression (concentrations in a single layer normalized per unit of the volume):


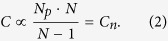


By approximating this model to actual physical conditions, we fix the distance between the particles to the minimal one, namely to the particle diameter, which is a small value compared with the longitudinal and transverse dimensions. In the limiting case, the entire volume is filled with the particles randomly and without gaps. Therefore, a condition must be entered:


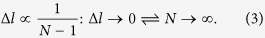


However, it should be mentioned that in the case when the distances between the layers Δ*l* are comparable with the particle diameter *D*, the volume has no gaps along the longitudinal coordinate. If Δ*l* < *D*, two particles may possibly ‘stick’ when simulation of the volume is performed. This situation leads to a new type and shape of the particle. If we impose an additional condition on the particles’ coordinates to prevent them from sticking together, the distribution will cease to be chaotic, since each new layer will depend on the previous one. For sufficiently large longitudinal size of investigated volume, the finite size of Δ*l* can be neglected and it can be considered infinitely small. With the addition of new conditions let us rewrite (2):


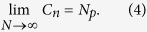


In our model, we build the dependencies of the CC function parameter on the number of particles in each layer as a proportional limit of the particles’ volumetric concentration.

### Single particle parameterization

The particles we are considering in this model are ideal optically homogeneous spheres of the material characterized by certain transparency coefficient. For simplicity of calculations they can be reduced to the two-dimensional flat circles that are normal to the direction of the incident beam. The spherical shape of the particles implies that the circles have inhomogeneous transparency increasing from the center to the periphery according to the following law:


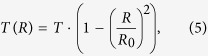


Here *R*_0_ is the particle radius, 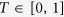 is the material transparency coefficient and 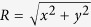. Thus we can alter transparency coefficient *T* achieving different absorptive capacity of the particles (see subsection General consideration of CC function dependencies through the numerical simulation in the Results and Discussion section).

### Wavefront propagation model

Consider the sequence of the optical simulation in detail. According to the simplified terms of a real random distribution, the particles are distributed in equal amounts merely in the certain planes of the volume. There are small equal distances Δ*l* between them. Mathematical model of the electromagnetic field passing through the volume with particles is explained below. Firstly, the beam with a plane wavefront from coherent laser source is simulated:


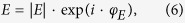


Here *E* is the complex amplitude and *φ*_*E*_ is the phase of incident beam. Important to note that hereinafter *E* = *E(x, y*) and *φ*_*E*_ = *φ*_*E*_(*x, y*) depend on Cartesian coordinates of *x* and *y*. Then we calculate the field that determines the distribution of particles in the first volume layer:


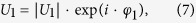


Here *U*_1_ is the complex amplitude and *φ*_1_ is the phase that set the distribution. Worth mentioning that in this paper, we confine consideration to the case of the absorbing particles, for which the relation *φ* = 0 is taking place. Multiplying the field *E* and the function that describes the particles in the first layer, we obtain the field after the layer:


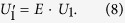


Using the angular spectrum (AS) of plane waves as the wavefront propagation method, we calculate the field propagation between the two adjacent layers distant from each other by the fixed distance Δ*l*. In accordance with this method, any diffracted field 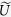 in the plane (*x, y*) located at the distance Δ*l* from the plane (*x*′, *y*′) where the wavefront *U(x*′, *y*′) is given, can be calculated by means of the following equation:


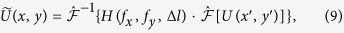


where *H(f*_*x*_, *f*_*y*_, Δ*l*) is the transfer function:


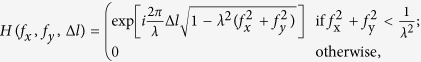



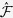
 and 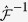 are direct and inverse Fourier transform operators, *f*_*x*_ and *f*_*y*_ are the spatial frequencies. For brevity we can write the equation for propagated wavefront at the distance Δ*l* in the operator format:


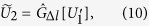


Here 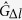 is an operator, which applies the AS method for the distance of Δ*l*, and 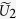 is the calculated diffracted field near the second layer. Then we consider the functions of the field that define the distributions of particles in the next layers: 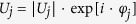, where *j* is a number of the layer. Finally, we can obtain a recurrence relation for the field passes through the volume with these layers:


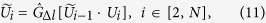


where *N* is a total number of layers in the volume. As a result, basing on the Eq. (11), we obtain the final field right after the last layer. This field is the result of interaction of the source field with all layers of the volume:


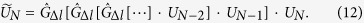


Then we simulate a propagation of the field from the last layer to the registration plane for the distance of *l*, yielding a hologram *I*_*H*_, which gives the necessary information about the object:


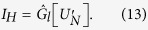


Sampling conditions restrict the maximum permissible longitudinal distance *l*_0_ for the calculation via AS method^43^, if pixel sizes Δ*x* = Δ*y*:


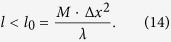


Here *M* is a number of pixels in the registration plane.

The resulting hologram is then numerically back propagated to the volume to obtain the images of two consecutive layers:


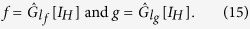


Here *l*_*f*_ and *l*_*g*_ are the distances from registration plane to the adjacent layers *f* and *g*, respectively. Thus, the images with focused particles therein are obtained and then compared by means of the CC function.

The further from the observer are the compared layers, the greater is the CC function error. The error occurs due to several reasons. The presence of the coherent background of unfocused particles makes a significant contribution to the error. This coherent background suppresses focused ones while moving deeper into the volume. The CC function thus ceases to distinguish the particles, because in fact identical images are compared. Therefore the *p*/*b* ratio of the CC function remains the same. The second error is related to the AS method approximation that was used in numerical simulation. This error is associated with the limited number of pixels in the image, as well as the certain number of gray scales for one pixel. In terms of real numerical models it is problematic enough to undergo the infinite limit (see Distribution of particles subsection). It also contributes to the possible error in the CC function calculation. Since that it is expedient to choose one pair of layers near to the observer.

### Image processing

In this subsection we describe the CC function and perform the identification of *p*/*b* ratio. In order to find the total concentration of particles in the volume after obtaining of two focused images of the adjacent layers *f* and *g* we apply the method that uses calculation of the CC function, defined as:


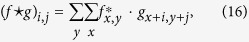


Here *x* and *y* are index numbers that reveal coordinates of the point where CC function calculation is performed, *i* and *j* are the shift-steps in the *x* and *y* directions and sign 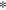 indicates a complex conjugation. In the case of faster computation using fast Fourier transform algorithm^44^ CC function can be calculated as:


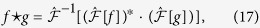


Here *f* and *g* are two-dimensional arrays.

**Figure 12:**
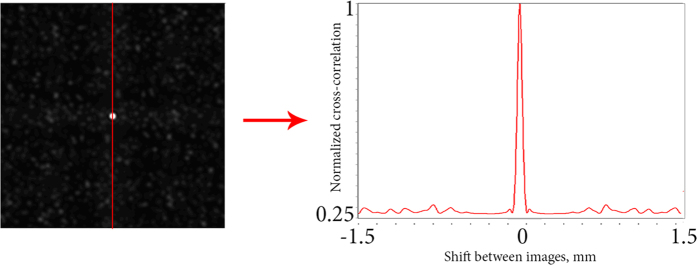
Transition from normalized 3D CC function function to its 2D section. The cut place is indicated.

Without loss of information, we can analyze the plane section of the CC function. Due to the symmetry relative to the optical axis of the studied distributions and of the particles themselves, CC function turns to be symmetrical as well. In this case, the parameters of CC function can be determined much faster. The transition is illustrated in Fig. 12.

**Figure 13:**
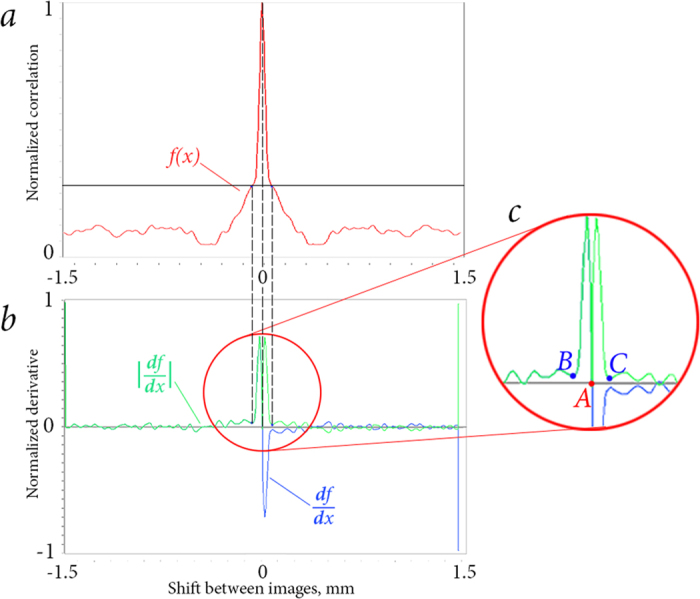
Correlation function section (**a**), its derivative and absolute valued derivative (**b**), larger consideration of the absolute derivative first minimum (**c**): *A* – null derivative, *B* and *C* – absolute derivative first minimum points.

By the definition, the origin of the CC function basement is the point of inflection of the CC function. Let us consider the absolute valued CC function derivative, which is calculated for positive and negative shift directions. Zero minimum corresponds to the peak, because the CC function reaches its maximum value at this point. The first minimum in both directions corresponds to the point of inflection of the CC function. The typical form of the CC function, its first derivative, and the definition of the basement boundaries are shown in Fig. 13.

After basement determination, *p*/*b* ratio is calculated. Based on the behavior of the CC function of two similar images, we assumed that *p*/*b* ratio depends on the particle concentration in a particular way (see Results and Discussion section).

## Conclusions

In this paper, we have given a discussion of methods, which are used to study the suspensions of particles in the volume, their application, and the use of the CC function in scientific and technical applications. We have proposed a new method for the study of such distributions. Its advantage is that it is based on calculation of the CC function, which allows rapid, down to the real time, estimation of the changes in the particles’ concentrations with other parameters of the system, such as the size and shape of the particles, remaining stationary. The proposed method requires pre-registration of several inline holograms for the calibration via numerical simulation. This operation is performed only once, if the size or shape of the particles is not being changed. Then by recording of the hologram of particles we can quickly assess their concentration in the certain layer, or in the whole volume, if the particles are distributed equiprobably.

After a general description of the model of the volume with particles and its study using inline digital holography and CC function, the basic theoretical aspects of our research were discussed and explained. To evaluate the applicability of our model in real physical experiment, we followed the properties of the CC function. After collecting of the basic dependencies of the CC function *p*/*b* parameter, namely the peak-to-basement ratio, on the normalized concentrations of particles in the volume, we found that they follow a linear law. In addition we have performed the analysis on the effect of several mixed parameters on the CC function peak-to-basement ratio and found that the particle concentration has dominant influence on the *p*/*b* ratio. Finally, we conducted several experiments, and by simulating the experimental conditions, we superimposed the results of the experiments on the model dependencies. A unique relation of experimental data and simulation was detected. We also discussed some directions for further development of the method, which we consider promising in connection with the various practical applications.

## Additional Information

**How to cite this article:** Vovk, T. A. and Petrov, N. V. Correlation Characterization of Particles in Volume Based on Peak-to-Basement Ratio. *Sci. Rep.* **7**, 43840; doi: 10.1038/srep43840 (2017).

**Publisher's note:** Springer Nature remains neutral with regard to jurisdictional claims in published maps and institutional affiliations.

## Figures and Tables

**Table 1:** Average dependency of *p*/*b* ratio on concentration and diameter of particles.

*p*/*b*	Diameter, *D* (mm)		
**Concentration**, *N*_*p*_	**0**.**054**	**0**.**12**	**0**.**18**
**5**	1.08	1.11	1.12
**50**	5.59	5.78	5.87
**100**	9.11	9.11	9.09

Parameters: *N* = 10, *T* = 0%, sizes of the screen 3.072 × 3.072 *mm*^2^, *λ* = 632.8 nm. CC function is calculated for the first and second layers.

**Table 2:** Average dependency of *p*/*b* ratio on concentration and transparency of particles.

*p*/*b*	Transparency, *T*		
**Concentration**, *N*_*p*_	**0**%	**10**%	**50**%
**5**	1.27	1.09	1.01
**50**	2.7	1.12	1.01
**100**	3.2	1.28	1.02

Parameters: *N* = 10, *D* = 0.054 mm, sizes of the screen 3.072 × 3.072 *mm*^2^, *λ* = 632.8 nm. CC function is calculated for the first and second layers.

**Table 3:** Average dependency of *p*/*b* ratio on diameter and transparency of particles.

*p*/*b*	Diameter, *D* (mm)		
**Transparency**, *T*	**0**.**054**	**0**.**12**	**0**.**18**
**0**%	1.27	2.34	3.08
**10**%	1.04	1.07	2.46
**50**%	1.01	1.01	1.01

Parameters: *N* = 10, *N*_*p*_ = 5, sizes of the screen 3.072 × 3.072 *mm*^2^, *λ* = 632.8 nm. CC function is calculated for the first and second layers.

## References

[b1] Erickson J. S., Hashemi N., Sullivan J. M., Weidemann A. D. & Ligler F. S. *In Situ* Phytoplankton Analysis: There’s Plenty of Room at the Bottom. Anal. Chem. 2, 839–850 (2012). 10.1021/ac201623k 21755976

[b2] Turpin B. J., Saxena P. & Andrews E. Measuring and simulating particulate organics in the atmosphere: problems and prospects. Atmos. Environ. 34, 2983–3013 (2000).

[b3] Ramanathan V. Aerosols, Climate, and the Hydrological Cycle. Science 294, 2119–2124 (2001). 11739947 10.1126/science.1064034

[b4] Cao X., Liu J., Jiang N. & Chen Q. Particle image velocimetry measurement of indoor airflow field: A review of the technologies and applications. Energ. Buildings 69, 367–380 (2014).

[b5] Zhang H., Chon C. H., Pan X. & Li D. Methods for counting particles in microfluidic applications. Microfluid. Nanofluid. 7, 739–749 (2009). 10.1007/s10404-009-0493-7 PMC7088397 32214956

[b6] Muste M., Yu K., Fujita I. & Ettema R. Two-phase versus mixed-flow perspective on suspended sediment transport in turbulent channel flows. Water Resour. Res. 41 (2005).

[b7] Soid S. & Zainal Z. Spray and combustion characterization for internal combustion engines using optical measuring techniques – A review. Energy 36, 724–741 (2011).

[b8] Zhang Y., Zhang Y., Qian Z., Ji B. & Wu Y. A review of microscopic interactions between cavitation bubbles and particles in silt-laden flow. Renew. Sust. Energ. Rev. 56, 303–318 (2016).

[b9] Brandenburg B. & Zhuang X. Virus trafficking–learning from single-virus tracking. Nature Rev. Microbiol. 5, 197–208, doi: 10.1038/nrmicro1615 (2007). 17304249 PMC2740720

[b10] Westerweel J. Fundamentals of digital particle image velocimetry. Meas. Sci. Technol. 8, 1379–1392 (1997).

[b11] Lee S. J. & Kim S. Advanced particle-based velocimetry techniques for microscale flows. Microfluid. Nanofluid. 6, 577–588 (2009).

[b12] Lindken R., Rossi M., Große S. & Westerweel J. Micro-Particle Image Velocimetry (micro-PIV): Recent developments, applications, and guidelines. Lab Chip 9, 2551 (2009). 19680579 10.1039/b906558j

[b13] Wereley S. T. & Meinhart C. D. Recent Advances in Micro-Particle Image Velocimetry. Annu. Rev. Fluid Mech. 42, 557–576 (2010).

[b14] Katz J. & Sheng J. Applications of Holography in Fluid Mechanics and Particle Dynamics. Annu. Rev. Fluid Mech. 42, 531–555 (2010).

[b15] Scarano F. Tomographic PIV: principles and practice. Meas. Sci. Technol. 24, 012001 (2013).

[b16] Gamble J. F., Tobyn M. & Hamey R. Application of Image-Based Particle Size and Shape Characterization Systems in the Development of Small Molecule Pharmaceuticals. J. Pharm. Sci. 104, 1563–1574 (2015). 25690940 10.1002/jps.24382

[b17] Buzalewicz I., Kujawińska M., Krauze W. & Podbielska H. Novel perspectives on the characterization of species-dependent optical signatures of bacterial colonies by digital holography. PloS one 11, e0150449 (2016). 26943121 10.1371/journal.pone.0150449 PMC4778909

[b18] Memmolo P. . Recent advances in holographic 3D particle tracking. Adv. Opt. Photonics 7, 713 (2015).

[b19] Saxton M. J. Single-particle tracking: connecting the dots. Nat. Methods 5, 671–672 (2008). 18668034 10.1038/nmeth0808-671

[b20] Yu X., Hong J., Liu C. & Kim M. K. Review of digital holographic microscopy for three-dimensional profiling and tracking. Opt. Eng. 53, 112306 (2014).

[b21] Genovesio A. . Multiple particle tracking in 3-d+ t microscopy: method and application to the tracking of endocytosed quantum dots. IEEE Trans Image Process 15, 1062–1070, doi: 10.1109/TIP.2006.872323 (2006). 16671288

[b22] Voronetskii A. V., Mikhailov V. N., Petrov N. V. & Stasel’ko D. I. Measuring the spatiotemporal parameters of motion of self-luminous particles in high-temperature supersonic flow. J. Opt. Technol. 79, 12–16 (2012).

[b23] Nikolaeva T. Y. & Petrov N. V. Statistical study of coherent images of particles in the volume of optical medium. Proc. SPIE 9216, 921612-921612-8 (2014).

[b24] Nikolaeva T. Y. & Petrov N. V. Characterization of particles suspended in a volume of optical medium at high concentrations by coherent image processing. Opt. Eng. 54, 083101 (2015).

[b25] Luu L., Wang Z., Vo M., Hoang T. & Ma J. Accuracy enhancement of digital image correlation with b-spline interpolation. Opt. Lett. 36, 3070–3072 (2011). 21847163 10.1364/OL.36.003070

[b26] Farsad M., Evans C. & Farahi F. Robust sub-micrometer displacement measurement using dual wavelength speckle correlation. Opt. Express 23, 14960–14972 (2015). 26072852 10.1364/OE.23.014960

[b27] Fricke-Begemann T. Three-dimensional deformation field measurement with digital speckle correlation. Appl. Opt. 42, 6783–6796 (2003). 14661786 10.1364/ao.42.006783

[b28] Malov A. & Pavlov P. Determination of surface roughness parameters optically opaque parts by speckle using spiral beams. Computer Opt. 36, 365–370 (2012).

[b29] Petrov N., Bespalov V., Zhevlakov A. & Soldatov Y. I. Determining the velocity of an object in water, using digital speckle-photography. J. Opt. Technol. 74, 779–782 (2007).

[b30] Ismadi M.-Z. . Optimisation of a stirred bioreactor through the use of a novel holographic correlation velocimetry flow measurement technique. PloS one 8, e65714 (2013). 23776534 10.1371/journal.pone.0065714 PMC3679106

[b31] Goch G., Prekel H., Patzelt S., Faravashi M. & Horn F. Precise alignment of workpieces using speckle patterns as optical fingerprints. CIRP Ann. Manuf. Techn 54, 523–526 (2005).

[b32] Petrov N. V., Goryunov A. E. & Pavlov P. V. Investigation of interaction of structured illumination with random scattering media. Proc. SPIE 9205, 92050T–92050T (2014).

[b33] Dimla D. E. Sensor signals for tool-wear monitoring in metal cutting operations — a review of methods. Int. J. Mach. Tool Manu. 40, 1073–1098 (2000).

[b34] Beals M. J., Fugal J. P., Shaw R. A., Lu J., Spuler S. M. & Stith J. L. Holographic measurements of inhomogeneous cloud mixing at the centimeter scale. Science 350, 87–90 (2015). 26430119 10.1126/science.aab0751

[b35] Shaw R. A. Particle-turbulence interactions in atmospheric clouds. Annu. Rev. Fluid Mech. 35, 183–227 (2003).

[b36] Park K. S. & Shin H. W. Studies on phyto-and-zooplankton composition and its relation to fish productivity in a west coast fish pond ecosystem. J. Env. Biol. 28, 415 (2007). 17929759

[b37] Godhe A. . Intercalibration of classical and molecular techniques for identification of Alexandrium fundyense (Dinophyceae) and estimation of cell densities. Harmful Algae 6, 56–72 (2007).

[b38] Dyomin V. V., Olshukov A. S. & Dzyuba E. V. Digital holographic video for studies of plankton dynamics. Atmos. Ocean. Opt. 21, 951–956 (2011).

[b39] Shapiro H. M. Practical flow cytometry(John Wiley & Sons, 2005).

[b40] Gasol J. & Giorgio P. Using flow cytometry for counting natural planktonic bacteria and understanding the structure of planktonic bacterial communities. Sci. Mar. 64, 197–224 (2000).

[b41] Langehanenberg P., Kemper B., Dirksen D. & Von Bally G. Autofocusing in digital holographic phase contrast microscopy on pure phase objects for live cell imaging. Appl. Opt. 47, D176–D182 (2008). 18594573 10.1364/ao.47.00d176

[b42] Gao P. . Autofocusing of digital holographic microscopy based on off-axis illuminations. Opt. Lett. 37, 3630–3632 (2012). 22940972 10.1364/OL.37.003630

[b43] Petrov N. V. . Phase retrieval of THz radiation using set of 2D spatial intensity measurements with different wavelengths. Proc. SPIE 8281, 82810J–1 (2012).

[b44] Goodman J. Introduction to Fourier optics(McGaw-Hill Physical and Quantum Electronics Series, 1968).

